# A model-guided dissociation between subcortical and cortical contributions to word recognition

**DOI:** 10.1038/s41598-019-41011-9

**Published:** 2019-03-14

**Authors:** Mario Braun, Martin Kronbichler, Fabio Richlan, Stefan Hawelka, Florian Hutzler, Arthur M. Jacobs

**Affiliations:** 1 Centre for Cognitive Neuroscience, Universität Salzburg, Salzburg, Austria; 2 Allgemeine und Neurokognitive Psychologie, Freie Universität Berlin, Berlin, Germany; 3 Center for Cognitive Neuroscience Berlin, Berlin, Germany; 4 Dahlem Institute for Neuroimaging of Emotion, Berlin, Germany; 5 Neuroscience Institute, Christian-Doppler Medical Centre, Paracelsus Medical University, Salzburg, Austria

## Abstract

Neurocognitive studies of visual word recognition have provided information about brain activity correlated with orthographic processing. Some of these studies related the orthographic neighborhood density of letter strings to the amount of hypothetical global lexical activity (GLA) in the brain as simulated by computational models of word recognition. To further investigate this issue, we used GLA of words and nonwords from the multiple read-out model of visual word recognition (MROM) and related this activity to neural correlates of orthographic processing in the brain by using functional magnetic resonance imaging (fMRI). Words and nonwords elicited linear effects in the cortex with increasing BOLD responses for decreasing values of GLA. In addition, words showed increasing linear BOLD responses for increasing GLA values in subcortical regions comprising the hippocampus, globus pallidus and caudate nucleus. We propose that these regions are involved in the matching of orthographic input onto representations in long-term memory. The results speak to a potential involvement of the basal ganglia in visual word recognition with globus pallidus and caudate nucleus activity potentially reflecting maintenance of orthographic input in working memory supporting the matching of the input onto stored representations by selection of appropriate lexical candidates and the inhibition of orthographically similar but non-matching candidates.

## Introduction

## Orthographic processing in visual word recognition

Successful processing of written words requires the activation and retrieval/reconstruction of stored orthographic information about these words from memory. On average, an English-speaking adult has an active vocabulary ranging from about 17000 to 45000 words^1^. The astonishing efficiency, speed and ease with which our brains usually carry out visual word recognition reveals indeed a fabulous faculty. This is all the more amazing given that all these words are formed by the combination of a limited number of symbols or letters implying a considerable orthographic similarity between words, which makes some letter combinations more familiar and easier to access and to remember than others^2^.

## Neighborhood density, global lexical activity, and familiarity

In studies of visual word recognition orthographic similarity is typically operationalized by neighborhood density (the number of orthographic word neighbors, which can be generated by changing one letter of a given word^3^). For example, when subjects make lexical decisions to words and nonwords the standard finding is that responses to words with a high number of neighbors are faster than to words with a low number of neighbors^4^. In contrast, reaction times to nonwords are slower when they have many word neighbors. This is suggested to be related to the high amount of global lexical activity (GLA) elicited by the activated word neighbors. However, according to the multiple read-out model of visual word recognition (MROM^5^) one of three response criteria are in effect when subjects make lexical decisions to words.

The response criteria are the following: (1) identification of words: if the activation of a lexical unit reaches a criterion (M-criterion) a YES-answer is produced and the word is identified; (2) a fast-guess mechanism: if GLA over all lexical units reaches a criterion (S-criterion) a YES-answer is elicited. GLA serves as a familiarity/word-likeliness estimate; (3) a time-out criterion (T-criterion) which is set to a fixed value. If this value is reached before the other two are reached a NO-answer will be given. The T- and the S-criteria vary depending on the GLA in the lexicon at a given point in time. If GLA is high then the T-criterion will be delayed and the S-criterion is set to a lower value resulting in an easier to reach deadline. Thus, each word is described by its specific activation over time and because there is multiple read-out from the model, a response can be caused by an item’s activation-match to these different criteria.

When processing words with a high number of neighbors it is assumed – as shown by computer simulations – that these generate high GLA values in the hypothetical mental lexicon through partial activation of all similar representation units^5^. This extra activity is supposed to allow for the fast-guess resulting in shorter ‘yes’ responses compared to words with only some neighbors and low values of GLA. As for words, nonwords with many neighbors are more wordlike and are supposed to elicit higher levels of GLA in the model suggesting the presence of words and thus to prolong processing of these items by shifting the temporal deadline. As GLA was previously shown to correlate with reaction times^6,7^ and to correlate with a number of linguistic measures like neighborhood density, word frequency, bigram frequency etc. (see Methods), we use GLA as a single computational measure of orthographic familiarity.

## Evidence from neuroimaging

By investigating responses to words and nonwords differing in number of neighbors, previous neuroimaging research found only little neural evidence for the GLA-hypothesis as implemented in the MROM. Rather, it was repeatedly found that blood oxygen level dependent (BOLD) responses were greater for less familiar stimuli (i.e., words with only some neighbors). For example, Binder and colleagues^8^ reported greater activation in response to words without neighbors in left angular gyrus and prefrontal and ventro-lateral temporal areas which was suggested to reflect the fact that accurate responses in lexical decisions depend on the activation of single word semantics. Moreover, Fiebach and colleagues^9^ found a lexicality by neighborhood density interaction in the left mid-dorsolateral prefrontal cortex and in a region slightly anterior to the pre-SMA in the medial superior frontal gyrus. Activity in the mid-dorsolateral prefrontal cortex was strongest in response to nonwords with many neighbors. In contrast, activation in the medial superior frontal gyrus was stronger for words with only some neighbors. Fiebach *et al*.^9^ interpreted this activation in frontal regions to reflect domain-general processing at a later post- or extra-lexical level rather than as reflecting activation in a hypothetical mental lexicon. However, recent findings showed that words with many neighbors elicited higher activity in the dorsolateral prefrontal cortex suggested to reflect working memory processes, and activity in the middle temporal cortex was related to the identification of words from long term memory^10^.

While it is clear that for successful reading, orthographically similar words must be efficiently distinguished from each other, it is still not exactly understood how and where (i.e., in which neural networks) the whole process of word recognition is carried out. A lot of neuroimaging evidence suggests that the ventral occipital-temporal cortex hosting the visual word form area (VWFA) is involved in the identification of sublexical and probably also of lexical codes^11–17^. Furthermore, evidence suggests an increasing specialization from letters to words from posterior to anterior^17^. Recent research indicates that visual word recognition is rather based on a highly distributed pattern of ‘orthographic’, ‘phonological’, and ‘semantic’ brain activity including the posterior inferior parietal lobule (angular and supramarginal gyrus), the lateral temporal cortex (middle and inferior temporal gyri), and parts of the left inferior frontal gyrus (pars opercularis, pars orbitalis and triangularis) rather than being restricted to a specialized region^8,18–21^.

## Subcortical involvement in memory for words

Previous research on visual word recognition mostly focused on cortical regions involving the dorsal and ventral reading pathways^22^. But there are also findings showing an involvement of subcortical regions, such as the perirhinal cortex and hippocampus^23–29^ and of the basal ganglia^30–35^.

A number of studies reported activity of the caudate nucleus of the basal ganglia during language tasks^30–40^. For example, stimulation of the left head of the caudate nucleus was found to induce perseveration in picture naming^40^ and showed higher activation in bilinguals when words had to be translated rather than repeated^41^. Caudate activity was also found when pictures had to be named in the non-dominant language or when subjects had to decide if a letter string is a word in the second language when it is also a word with a different meaning in the first^42^. Further evidence for an involvement in lexico-semantic control is provided by caudate activation in bilingual semantic judgment tasks^39^ and by studies showing activation of the caudate in the maintenance of visual and verbal information in working memory^30,43,44^.

## Recollection and familiarity

By many accounts^45–49^ the hippocampus is suggested to play an important role in recollection and to be involved in the formation of associative memories^50,51^. In line with this view are findings showing hippocampus activation during learning of new lexicons^23,52^ and results showing correlations of hippocampal grey matter volume with form-sound associations of words^53^. In contrast to its widely accepted role in recollection there is considerable disagreement about the hippocampus’ role in processing the familiarity of stimuli, which by most views is believed to be supported by the perirhinal cortex^48,54–56^. However, there is evidence in support of the idea that the hippocampus supports both recollection and familiarity^57–63^.

## The present study

Taken together, previous studies investigating lexical processing by manipulating orthographic similarity in visual word recognition tasks reported mixed results. Some studies found evidence for GLA-effects compatible with the activation of orthographically similar representations^6,7^ while others did not^8,9^.

Previous studies mostly used the lexical decision task to investigate visual word recognition, which potentially introduces extra-lexical executive processes thus making it difficult to dissociate lexical and extra-lexical processing. To avoid such potential confounds we used a silent reading paradigm. Furthermore, we presented only short words and nonwords (four letters in length) posing only low demands on the reading process itself.

As previously shown the task used has an influence on how stimuli will be processed^64^. Silent reading will probably result in less brain activity elicited by our stimuli because no explicit decision related process is involved and reading is a highly automated process. However, in terms of ecological validity, if we investigate processes of (silent) reading we should use silent reading as a task. Thus, with respect to previous results obtained with the lexical decision task, we would expect less brain activity in general and probably less decision-related brain activity in regions involved in such processing.

Thus, with the present fMRI study we aimed to investigate the neural bases of orthographic processing at a more fine-grained level by using model-generated GLA (which was shown to be correlated with reaction times) as an index of orthographic familiarity (instead of using a single linguistic measure such as, e.g., neighborhood density). For this, we used the GLA values of 300 words and 300 nonwords as simulated by the MROM^5^. We hypothesized that we should observe a systematic variation of BOLD responses to words and nonwords corresponding to these levels of GLA. GLA-correlated neural activation should provide information about orthographic familiarity processing in the brain by identifying brain regions which potentially could be parts of a distributed mental lexicon. In addition, if GLA is correlated with brain activity in subcortical regions like the hippocampus or basal ganglia we should observe a systematic graded variation of the BOLD response for different levels of GLA which could be interpreted as reflecting familiarity processing.

## Results

### Imaging results

#### Words and Nonwords compared to Baseline

**Figure 1:**
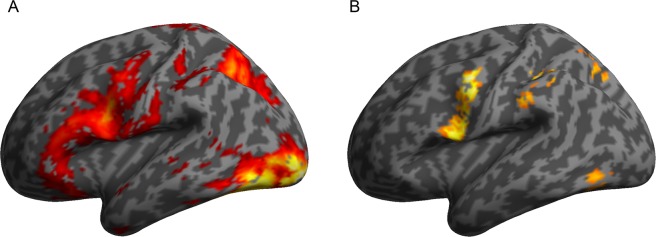
(**A**) BOLD response showing greater activation for words and nonwords compared to fixation baseline in fusiform gyrus, superior parietal lobule, angular gyrus, supramarginal gyrus, precentral gyrus, pars opercularis, SMA, cingulate cortex, hippocampus, on whole-brain level (FWE corrected, p < 0.05, cluster extent 25 voxels). (**B**) BOLD response showing greater activation for nonwords compared to words in supramarginal gyrus, angular gyrus, superior parietal lobule, precentral gyrus, precuneus, occipital fusiform gyrus and middle temporal gyrus on whole-brain level (cluster-level FDR corrected, p < 0.05, voxel-level uncorrected, p < 0.001).

We first contrasted words and nonwords with the fixation baseline at the whole-brain level (FWE corrected, p < 0.05, cluster extent 25 voxels). Both words and nonwords recruited nearly the same regions showing activation in regions of the ventral stream, including the occipital pole, the occipital fusiform/temporal cortex and the temporal gyrus. Furthermore, words and nonwords showed higher activation in the superior parietal lobule/anterior parietal sulcus including parts of the angular and supramarginal gyri as well as in the precentral gyrus and the pars opercularis of the inferior frontal gyrus. Another cluster was evident for words and nonwords in premotor cortex/supplementary motor area. In addition, higher activation compared to baseline was obtained in bilateral hippocampus and in the central/postcentral gyrus. Furthermore, the central opercular cortex/postcentral gyrus showed higher activation for nonwords compared to the fixtion baseline (see Tables 1 and 2 and Fig. 1).

**Table 1:** Brain regions showing higher activation for words compared to fixation baseline (FWE corrected, p < 0.05, cluster extent 25 voxels).

*Brain Region*	*BA*	*Hem*	*x*	*y*	*z*	*cluster size*	*Zmax*
**words** > **fixation**							
Occipital pole, lateral occipital cortex, occipital fusiform cortex, temporal occcipital fusiform cortex	18	L	−21	−97	−11	3307	>8
			−45	−64	−17		
Superior parietal lobule, angular Gyrus, posterior supramarginal gyrus	7	R	−24	−58	40	314	>8
Precentral gyrus, pars opercularis	44	L	−42	2	25	674	>8
Supplementary motor area, cingulate gyrus, parcingulate gyrus	6	L	−3	5	49	66	7.19
Hippocampus, amygdala		R	18	−10	−23	62	7.19
Anterior/posterior temporal fusiform Cortex, hippocampus	20	L	−33	−10	−41	41	6.21
			−33	−19	−29		
Pre-/postcentral gyrus,	4/6	L	−3	−31	70	226	6.21
Posterior parahippocampal gyrus, posterior temporal fusiform cortex		R	21	−31	−17	32	5.98
Hippocampus, amygdala	—	L	−18	−16	−17	121	5.60

Note. x, y, z = peak coordinates according to MNI stereotactic space, cluster size in voxels, BA = Brodmann Area, Hem = Hemisphere.

**Table 2:** Brain regions showing higher activation for nonwords compared to fixation baseline (FWE corrected, p < 0.05, cluster extent 25 voxels).

*Brain Region*	*BA*	*Hem*	*x*	*y*	*z*	*cluster size*	*Zmax*
***nonwords*** > ***fixation***							
Occipital pole, lateral occipital cortex, occipital fusiform cortex, temporal occcipital fusiform cortex	18	L	−21	−97	−11	2847	>8
			−45	−64	−17		
Superior parietal lobule, angular Gyrus, posterior supramarginal gyrus	7	R	−27	−61	46	643	>8
Precentral gyrus, pars opercularis	44	L	−42	2	25	990	>8
Supplementary motor area, cingulate gyrus, parcingulate gyrus	6	L	−6	5	52	126	>8
Hippocampus, amygdala		R	15	−10	−20	105	6.71
Opercular cortex, postcentral gyrus, parietal operculum, anterior supramarginal gyrus, anterior division, planum temporale	20	L	−60	−19	19	63	6.33
Anterior/posterior temporal fusiform Cortex, hippocampus	20	L	−33	−10	−41	70	5.99
			−15	−16	−20		
			−33	−7	−23		
Pre-/postcentral gyrus,	4/6	L	−9	−22	73	58	5.66

Note. x, y, z = peak coordinates according to MNI stereotactic space, cluster size in voxels, BA = Brodmann Area, Hem = Hemisphere.

#### Words vs. Nonwords

The direct comparison of words and nonwords (cluster-level FDR corrected for multiple comparisons, p < 0.05) revealed that no region was activated higher for words than for nonwords. In contrast, several left hemispheric regions comprising the anterior and posterior supramarginal gyrus, the precentral gyrus, the superior parietal lobule, the inferior occipital cortex and the occipital fusiform gyrus showed greater activity for nonwords compared to words (Fig. 1).

#### Effects of GLA for Words and Nonwords

To identify regions related to orthographic similarity we looked for effects of GLA by contrasting the highest vs. lowest levels of GLA, separately for words and nonwords. We looked also for effects with GLA as a continous variable on the whole brain level. The regions and the effects for words and nonwords were nearly the same as for the reported analyses with GLA-levels. The results of this analysis can be found as Supplementary Table S1.

For nonwords no region showed higher activity for high GLA- than for low GLA-nonwords. In contrast, low GLA-nonwords showed greater activity than high GLA-nonwords in many regions comprising the pre- and postcentral gyrus and the pars opercularis of the inferior frontal gyrus (−57 2 22), left superior parietal lobule, left anterior/posterior supramarginal gyrus, left angular and postcentral gyrus (−36 −43 40), left planum polare, Heschl’s Gyrus, left anterior superior temporal gyrus, left central opercular cortex (−51 −1 −5), right superior lateral occipital cortex, right angular gyrus, right precuneus (27 − 61 34), left lateral occipital cortex (−33 −88 13), visual cortex, left supplementary motor area (−3 −1 58), left lingual gyrus, precuneus (−3 −67 7) and right anterior/posterior supramarginal gyrus, right superior parietal lobule, right angular and postcentral gyrus (45 −40 55).

**Figure 2:**
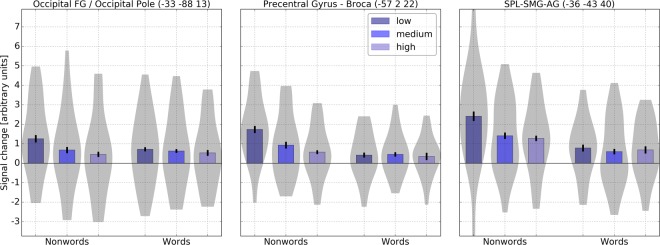
Signal change in response to words and nonwords showing increasing linear activation for decreasing levels of GLA for nonwords but not for words in the precentral gyrus, superior parietal lobule, supramarginal and angular gyri, planum polare and occipital fusiform gyrus. (whole-brain level, voxel-level uncorrected, p < 0.005, cluster extent threshold 25).

We calculated 2 (lexical status: words/nonwords) × 3 (GLA: low/medium/high) repeated measures ANOVAs and tested for linear effects of GLA. All regions showed main effects of GLA (all Fs > 3.2) and all of these were found to be linear (all Fs > 8.5). Furthermore, the left dorsolateral prefrontal cluster (F(2, 38) = 8.729, p = 0.001), left planum polare (F(2, 38) = 5.373, p = 0.009), right lateral superior occipital cortex/angular gyrus (F(2, 38) = 6.179, p = 0.005) and left lingual gyrus (F(2, 38) = 4.358, p = 0.020) showed interactions of lexical status and GLA which was due to linear effects of GLA for nonwords but not for words in these regions (see Table 3 and Fig. 2).

**Table 3:** Brain regions showing higher activation for nonwords with high GLA compared to nonwords with low levels of GLA and vice versa (voxel-level uncorrected, p < 0.005, cluster extent threshold 25).

*Brain Region*	*BA*	*Hem*	*x*	*y*	*z*	*cluster size*	*Zmax*
**high GLA-nonwords** > **low GLA-nonwords**							
—	—	—	—	—	—	—	—
**low GLA-nonwords** > **high GLA-nonwords**							
Pre- and postcentral gyrus, pars opercularis	6/44/48	L	−57	2	22	231	4.76
Superior parietal lobule, supramarginal/angular gyrus and postcentral gyrus	7/39/40	L	−36	−43	40	160	4.01
Planum polare, heschl’s gyrus, anterior superior temporal gyrus	48	L	−51	−1	−5	32	3.69
Superior lateral occipital cortex, angular gyrus, precuneus	19	R	27	−61	34	84	3.62
Inferior lateral occipital cortex, occipital fusiform gyrus, occipital pole	18	L	−33	−88	13	56	3.54
Supplementary motor area	6	L	−3	−1	58	62	3.47
Lingual gyrus, intracalcarine cortex, precuneus	17	L	−3	−67	7	27	3.34
Posterior supramarginal gyrus, superior parietal lobule, angular gyrus, postcentral gyrus, anterior supramarginal gyrus	40	R	45	−40	55	25	2.99

Note. x, y, z = peak coordinates according to MNI stereotactic space, cluster size in voxels.

The contrast of higher activity for high GLA-words compared to low GLA-words showed the strongest activity in subcortical regions comprising the hippocampus and parts of the basal ganglia. The strongest activation was found for a globus pallidus/hippocampus cluster. Furthermore, the caudate tail and body showed stronger activation in response to high GLA-words. In addition, four cortical regions showed higher activity for high GLA-words compared to low GLA-words comprising, right posterior cingulate cortex, left supramarginal gyrus and right insula (see Table 4). To verify if regions associated with higher activity for high GLA compared to low GLA stimuli were preferentially associated with words or nonwords we calculated 2 (lexical status: words/nonwords) by 3 (GLA: low/medium/high) repeated measures ANOVAs on the peak values of the respective contrasts and checked for linear effects of GLA.

**Table 4:** Brain regions showing higher activation for words with high GLA compared to words with low GLA and vice versa (voxel-level uncorrected, p < 0.005, cluster extent threshold 25).

*Brain Region*	*BA*	*Hem*	*x*	*y*	*z*	*cluster size*	*Zmax*
**high GLA-words** > **low GLA-words**							
Globus pallidus/Hippocampus	—	L	−15	−7	1	78	5.02
	—	L	−21	−13	−14		
Caudate nucleus (tail)	—	L	−18	−46	13	42	4.03
Fornix	—	R	21	−34	13	87	3.53
Caudate nucleus (body)	—	L	−18	−13	22	28	3.49
Posterior cingulate cortex	25	R	15	−19	37	27	3.44
Anterior supramarginal gyrus	40	L	−27	−37	34	33	3.42
Insula	13	R	36	−1	−14	52	3.24
**low GLA-words** > **high GLA-words**							
Lateral occipital and occipital fusiform cortex	19	L	−30	−82	−2	27	4.19
Pre-motor cortex	6	L	−18	−4	64	32	3.95
Superior parietal lobule, posterior supramarginal gyrus, primary somatosensory cortex	40	L	−39	−40	58	43	3.78

Note. x, y, z = peak coordinates according to MNI stereotactic space, cluster size in voxels.

The ANOVA for the globus pallidus/hippocampus cluster (−15 −7 1/−21 −13 −14) revealed a main effect of GLA (F(1, 19) = 6.386, p = 0.004) and an interaction of lexical status with GLA (F(2, 38) = 4.214, p = 0.022). The main effect of GLA was linear (F(1, 19) = 9.962, p = 0.005). Closer evaluation of the interaction showed that the effect of GLA was significant for words (F(2, 38) = 6.111, p = 0.005) and nonwords F(2, 38) = 4.555, p = 0.017). However, the GLA effect was linear for words (F(1, 19) = 12.236, p = 0.002), but not for nonwords (F(1, 19) = 1.286, p = 0.271).

**Figure 3:**
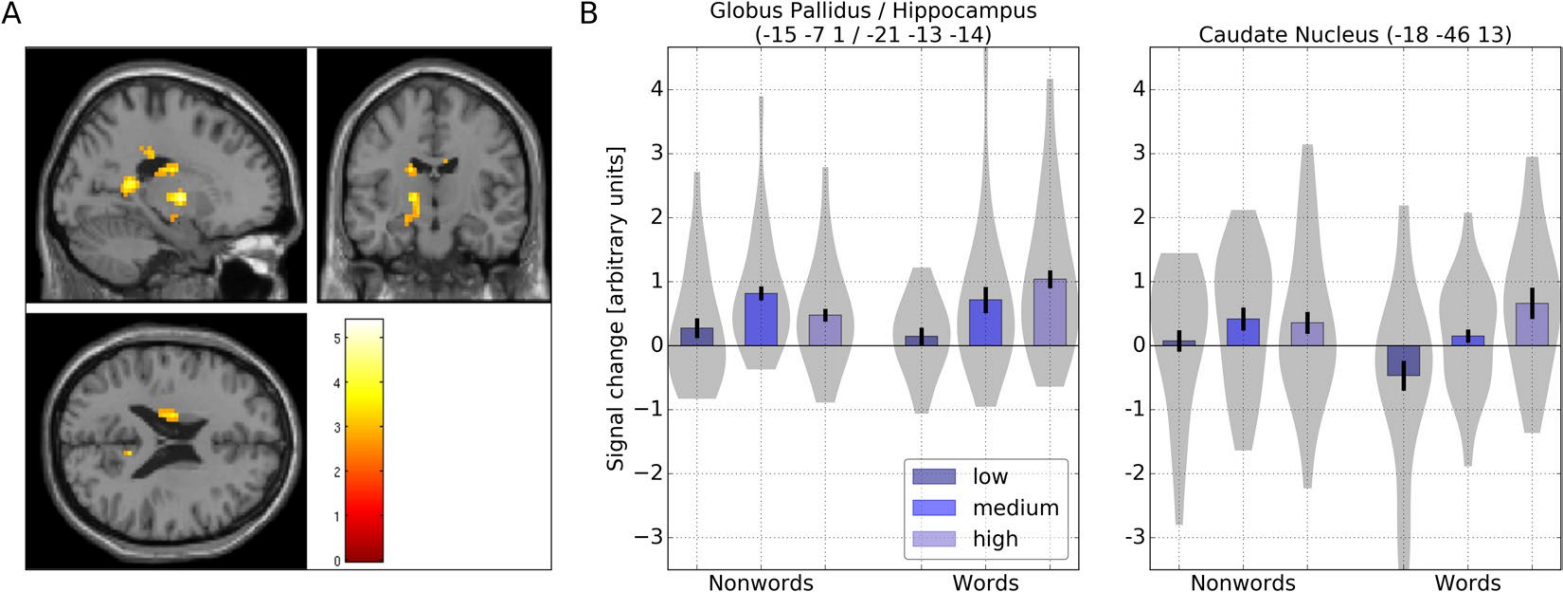
(**A**) BOLD response for words and nonwords in caudate nucleus and globus pallidus/hippocampus showing increasing linear effects for increasing levels of GLA for words but not for nonwords on the whole-brain level. (**B**) Signal change in response to words and nonwords with increasing linear activation for increasing levels of global lexical activity in the globus pallidus/hippocampus and caudate nucleus for words but not for nonwords on whole-brain level (voxel-level uncorrected, p < 0.005, cluster extent threshold 25).

Furthermore, the tail of the left caudate nucleus (−18 −46 13) showed a main effect of GLA (F(1, 19) = 3.914, p = 0.028) and the interaction of lexical status with GLA (F(2, 38) = 2.940, p = 0.065) approached significance. The main effect of GLA was again linear (F(1, 19) = 5.106, p = 0.036). The evaluation of the interaction showed that the effect of GLA was significant for words (F(2, 38) = 5.510, p = 0.008), but not for nonwords (F(2, 38) = 0.839, p = 0.440). Again, the test for linear effects of GLA was significant for words (F(1, 19) = 5.993, p = 0.024), but not for nonwords (F(1, 19) = 1.293, p = 0.270) (see Table 4 and Fig. 3).

Further main effects of GLA were found in the caudate body (F(2, 38) = 4.285, p = 0.021), the right posterior cingulate cortex (F(2, 38) = 6.084, p = 0.005), and the left anterior supramarginal gyrus (F(2, 38) = 4.688, p = 0.015), which were also linear: caudate body (F(1, 19) = 9.221, p = 0.007), right posterior cingulate (F(1, 19) = 12.260, p = 0.002), left anterior supramarginal gyrus (F(1, 19) = 8.151, p = 0.010). These regions showed no main effect of lexical status and no interaction of lexical status and GLA suggesting that orthographic similarity was not processed differently for words and nonwords in these regions.

**Figure 4:**
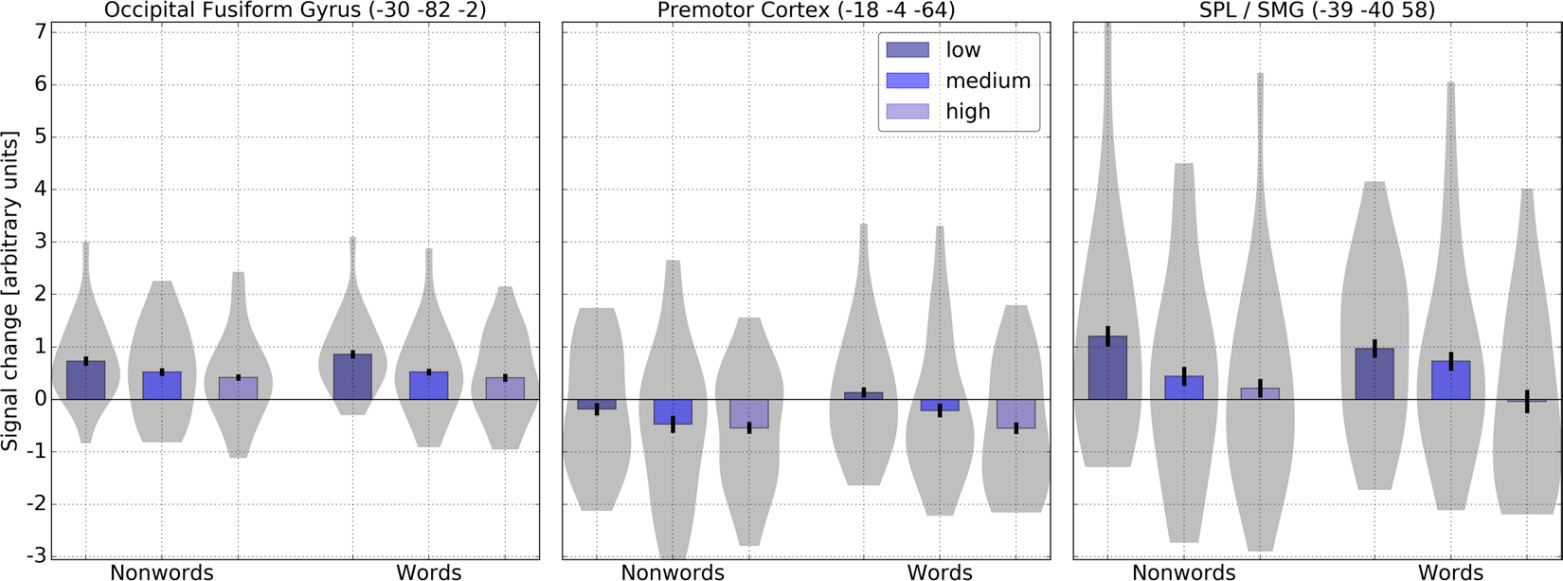
Signal change for words and nonwords in regions showing increasing activation for decreasing levels of GLA in left inferior lateral occipital and occipital fusiform cortex, premotor cortex and superior parietal lobule on whole-brain level (voxel-level uncorrected, p < 0.005, cluster extent threshold 25).

The reverse contrast of higher activity for low GLA-words compared to high GLA-words revealed significant differences in left inferior lateral occipital and occipital fusiform gyrus (−30 −82 −2), the superior frontal cortex/precentral gyrus (−18 −4 64) and in the superior parietal lobule/posterior supramarginal gyrus/primary somatosensory cortex (−39 −40 58) (Table 4 and Fig. 4). Thus, in regions believed to be involved in orthographic-phonological conversion and articulatory processing. The repeated measures ANOVAs and the linear contrasts revealed higher activity for low GLA-words in all three regions: occipital fusiform (F(2, 38) = 8.390 p = 0.001; F(1, 19) = 12.924, p = 0.002), premotor cortex (F(2, 38) = 6.313, p = 0.004; F(1, 19) = 15.776, p = 0.001) and superior parietal lobule/posterior supramarginal gyrus (F(2, 38) = 12.644, p < 0.001; F(1, 19) = 40.548, p < 0.001).

## Discussion

The present fMRI study aimed to investigate the neural basis of orthographic processing by using GLA as the simulated activity of a computational model of visual word recognition of words and nonwords as an index of orthographic familiarity and similarity. We reasoned that if in response to these words and nonwords differential GLA-correlated neural activation is found in brain regions believed to be involved in visual word processing, (e.g., ventral occipital-temporal cortex), or in regions suggested to process orthographic-phonological relations, (e.g., inferior parietal, inferior frontal and middle temporal cortex), or in subcortical regions involved in recollection and/or familiarity processing, (e.g., hippocampal complex or parts of the basal ganglia), this would provide evidence for these regions to be involved in the processing of orthographic familiarity and similarity.

The words and nonwords in the present study activated the reading network of the left ventral stream in the occipital pole, the inferior occipital-temporal cortex, and the fusiform gyrus. Activation was also found in the dorsal stream where words and nonwords showed higher activation compared to fixation baseline in the superior parietal lobule, the anterior intraparietal sulcus, parts of the angular and supramarginal gyri, as well as in precentral gyrus and pars opercularis of the inferior frontal gyrus and premotor and supplementary motor area (see Fig. 1 and Tables 1 and 2).

To investigate orthographic processing more specifically, we looked for brain activity in response to model-generated GLA of words and nonwords assumed to reflect different levels of orthographic similarity. The results showed differential brain activity for the different levels of GLA. Nonwords with low GLA always elicited higher activity than nonwords with high GLA and this activity was found in regions comprising left pars opercularis of the inferior frontal gyrus, left pre- and postcentral gyrus, left superior parietal lobule (supramarginal and angular gyri), left superior/inferior lateral occipital cortex and the occipital pole (see Table 3 and Fig. 2).

In contrast to nonwords, responses to words showed a more differential pattern. As nonwords words elicited linear effects with increasing brain activity for decreasing levels of GLA in cortical regions comprising left inferior lateral occipital, occipital fusiform cortex and superior parietal lobule, as well as a decrease in deactivation with decreasing levels of GLA in the premotor cortex (see Table 4 and Fig. 3). One might speculate that this activity could be related to higher demands on the inhibition of button presses in response to more familiar words and word-like nonwords.

Thus, words and nonwords showed higher activity in response to less familiar items in regions suggested to be involved in identification, articulation and also in grapheme-phoneme conversion like the fusiform gyrus, pars opercularis, precentral gyrus and superior parietal lobule. This higher activity in response to lower levels of GLA is likley related to more effortful processing of these items.

However, words elicited also linear effects of increasing brain activity for increasing levels of GLA in subcortical regions comprising a globus pallidus/hippocampus cluster and the body and tail of the caudate nucleus (see Table 4 and Fig. 3).

Concerning the recognition of previously encountered stimuli dual-process theories of recognition memory assume that the processes of familiarity and recollection are in effect and to be supported by structures of the medial temporal lobe^64^. Most researchers agree that the hippocampus is involved in the recollection of memories^49,65,66^ whereas the perirhinal cortex is suggested to support familiarity processing^45,46,48^.

However, recent research suggests a possible strength confound when investigating recollection and familiarity based recognition memory. It was proposed that recollection based memories in previous studies were always stronger memories and familiarity based memories were always somewhat weaker and thus the obtained activation differences in hippocampal and perirhinal regions could simply have reflected differences in the degree of strength and not in kind^60^. In line with this view, recent studies reported increased hippocampus activation for strong familiarity based memories^57,58,61^ adding support for familiarity processing in the hippocampus. Activity in the hippocampus and perirhinal cortex during learning varied as a function of subsequent item memory strength when source memory strength was held at chance level, suggesting that activation in the medial temporal cortex signals memory strength^57^. Furthermore, a non-linear relationship between predictive memory strength in the hippocampus and perirhinal cortex was found^58^. Both regions showed a differential pattern of activity for items with high vs. medium and medium vs. low memory strength. A cluster in left hippocampus discriminated between high and medium memory strength and a cluster in right perirhinal cortex discriminated between medium and low memory strength, suggesting that hippocampus activation signals strong memories and perirhinal cortex somewhat weaker memories. Furthermore, results from intracranial EEG showed that the hippocampus is found to be active for both recollection and familiarity^67^.

In line with the above reported findings and the idea that the hippocampus could also signal the familiarity of stimuli^68^, we would like to propose that the linear increase in BOLD response for words with increasing GLA in the globus pallidus/hippocampus cluster reflects the (pre-)activation of word representations with similar orthographic codes in long-term memory. High GLA-words are likely to be encountered more often and to have more neighbors with higher frequencies and also higher bigram counts and frequencies, as was the case in the present study (see Table 5). Thus, they are per se more similar to many other word representations and thus more familiar. We suggest that this resulted in the formation of stronger memory traces for orthographically similar words, which are activated when high GLA-words are encountered. A higher activity for high GLA-words could thus reflect the matching process of word stimuli onto orthographic representations in long-term memory. The specifics of this matching process probably depend on the level of orthographic similarity between the input and stored word representations in long-term memory, but probably also on inter-item similiarity of the stored representations. We thus suggest that higher activity in the hippocampus in response to high GLA-words reflects a higher degree of familiarity with the input as compared to low GLA-words. Such an interpretation would be in line with the assumption of the declarative/procedural model of memory^28^, proposing the hippocampus to be part of a declarative memory system and to be involved in the storage of lexico-semantic information. In addition, it is likely that perception of high GLA-words not only leads to stronger activation of representations of orthographically similar words, but also to stronger activation of other features of these words, such as phonological and semantic ones^26,69,70^.

**Table 5:** Stimulus means of important psycholinguistic measures for words and nonwords.

	GLA	LogF	Fmio	N	FN	BiC	BiF
Words-1	0.20	2.35	50.46	1.62	62.20	23.78	2231.02
Words-2	0.25	2.52	91.13	3.35	239.39	35.35	4442.91
Words-3	0.30	3.83	186.78	6.67	1383.58	48.64	9973.79
Nonwords-1	0.16	—	—	1.73	146.09	18.06	2130.37
Nonwords-2	0.21	—	—	3.45	374.84	31.46	3189.49
Nonwords-3	0.26	—	—	5.66	770.90	41.24	6238.52

Note. GLA = Global lexical Activity, LogF = log frequency, Fmio = word frequency per million, N = number of neighbors, FN = Summed frequency of the number of neighbors, BiC = Number of times the bigrams of a word do occur in other words, BiF = Summed frequency of words which share one or more bigrams.

Similar to words, nonwords elicited higher activity in the hippocampus compared to the fixation baseline (see Table 2). In contrast to words, nonwords showed no linear effect for different levels of GLA in the hippocampus, which contrasts with predictions of models of visual word recognition that assume similar linear effects for words and nonwords as a function of similar levels of GLA. One explanation for this dissociation could be that in addition to the activation of orthographically similar representations words activate their item-specific representation in memory which should boost activation for highly familiar stimuli^69^ compared to less familiar ones, probably also driven by semantic activity. In contrast, nonwords which by definition have no item-specific representations exactly matching the input (and no semantic representations) would fail to produce such item-specific activation, which in turn results in lower activation and no linear effects of GLA.

Concerning the activation of the globus pallidus and the caudate nucleus previous language studies studies showed a possible involvement in higher cognitive processing^30,39,70–74^. Increased caudate activity for semantic memories was reported during living/non-living decisions^70^. Furthermore, caudate nucleus activity was found for the type of semantic attributes during similarity judgments for written words^71^. In addition, caudate activity was found to be higher in response to words than to pseudowords in an auditory semantic priming study^72^ and for late-learned words compared to early-learned words^73^, as well as in high-level semantic ambiguity processing^74^. Furthermore, findings from intracranial electrical stimulation of the caudate nucleus and putamen during surgery for tumors were found to result in anarthria (putamen), whereas stimulation of the caudate nucleus led to perserverations suggesting putamen involvement in motor processing and caudate nucleus involvement in the cognitive control of language^39^. A recent resting-state functional connectivity study with 970 healthy subjects with seed regions in Broca’s and Wernicke’s areas also identified an extended network, which included not only prefrontal, temporal and parietal cortical regions, but also subcortical ones, such as the bilateral caudate and left putamen/globus pallidus and subthalamic nucleus which were found to be functionally connected to Broca’s and Wernicke’s areas^75^.

Most models of visual word recognition do not directly speak to brain regions involved in language processing but some models do and suggest an subcortical involvement. The lexical selection model allocates the selection of appropriate candidate representations to the basal ganglia^33,76^. The basal ganglia are proposed to contribute to the alignment of language related input into ongoing language plans by monitoring, inhibiting and selecting appropriate lexical candidates. Specifically, it is proposed that the basal ganglia are involved in a monitoring process, which becomes active in case too many lexical alternatives were activated and scanning for improper candidates is necessary to inhibit and preclude these items from further processing.

Moreover, Longworth and colleagues^34^ tested the idea of the declarative/procedural memory model^26^ that the basal ganglia are part of the fronto-striatal procedural memory system where grammatical rules are automatically applied to language. Contrary to the expectations, their results showed that dysfunction of the basal ganglia did not lead to impairment in grammatical processing, but instead to difficulties in suppressing semantically appropriate alternatives. In addition, patients with striatal dysfunctions were found to have difficulties to suppress the infrequent meaning of homophones in semantic priming studies providing further evidence for an involvement of the basal ganglia in the selection process of appropriate lexical candidates^76^.

Previous research showed an involvement of the basal ganglia and especially the caudate in articulatory aspects of language^77^. However, it seems that caudate activation is not restricted to language tasks involving a productive component and thus not solely to depend on the articulatory aspects of language, as evidenced by caudate activation during listening to auditorily presented narratives^78^, or during reading of texts with either positive or negative valence^79^.

A number of studies with healthy participants showed caudate nucleus and globus pallidus activation for visual and verbal material mainly during the maintenance phase of working memory tasks, but sometimes during encoding and retrieval^31,42–44,80^. Bilateral caudate nucleus showed prolonged activation during the maintenance phase for both materials and caudate head showed higher activation during retrieval in the verbal condition. Especially, the tail of the caudate nucleus showed more prolonged activation during verbal short-term memory^43^.

Moreover, equally active working memory load dependent activation of the left anterior caudate, left anterior putamen and globus pallidus was found during encoding and maintenance phases, but not during the response phase in a Sternberg task^31^ (for similar results see^81,82^ for globus pallidus activation during the response phase). These results were interpreted to reflect an important role of the anterior basal ganglia in working memory load dependent encoding, maintenance and retrieval. Such an interpretation is further in line with electrophysiological findings from primates^83,84^ and results of a meta-analysis based on human functional connectivity measures suggesting a tripartite division of the basal ganglia into motor, associative and limbic regions^80^. Thus, besides the well-established role of the basal ganglia in motor control much evidence suggests an involvement in language processing, most probably by supporting verbal working memory processes and the selection and inhibition of competing lexical alternatives.

In line with these findings we suggest that the linear activity in response to words in the globus pallidus and the caudate reflects an involvement in working memory and the selection and inhibition of lexical candidates during silent reading. Compared to low GLA- and medium GLA-words, high GLA-words in our study activate more orthographically similar representations posing a higher working memory load, higher maintenance effort during matching the input against stored representations, and higher effort for the selection of appropriate and the inhibition of inappropriate candidates, theoretically associated with the hippocampus.

In summary, the results showed that model simulated GLA – as a computational measure from a model of visual word recognition – is likely to reflect orthographic familiarity and wordlikeliness. Words and nonwords showed increased activation in response to decreasing levels of GLA in cortical regions suggested to be involved in the perception, articulation and grapheme-phoneme conversion. Probably reflecting more effortful processing of these items. In addition, the brain’s response to different levels of GLA of words showed that the more familiar a word the higher the subcortical activation in the basal ganglia and the hippocampus. We suggest that this activation reflects the level of familiarity with these words and probably to be based on the interaction of working memory and long-term memory processes in these regions.

## Method

We used the same procedure, experimental settings and the same scanner parameters as in^10^.

### Ethics

The study was approved by the ethics committee of the University of Salzburg (”Ethikkommission der Universität Salzburg”) and was in accordance with the principles expressed in the declaration of Helsinki. Informed consent was obtained from all participants.

### Participants

Twenty-three healthy subjects (eight men) participated in the fMRI study. All were right-handed, native German speakers. All had normal or corrected to normal vision, were free of any current or past neuropsychiatric disorders, and did not take psychoactive medication. All subjects were skilled readers with no reading disabilities. All subjects were not bilinguals in terms of having the same level of fluency in two or more languages. Mean age was 25.4 years (Range: 18–44; SD: 5.8). Three subjects were excluded from the analysis, two because of a programming error and one due to a hardware failure. Participants were recruited by students of the University of Salzburg and received course credit for their participation. All participants provided written informed consent and were tested individually at the Centre for Cognitive Neuroscience (CCNS) at the University of Salzburg.

### Procedure

Before the experimental session subjects performed a practice session with 20 items on a laptop outside the scanner. In the scanner, subjects were asked to read the words and nonwords silently. The scanning session lasted about 50 minutes and the whole experiment about one hour and 30 minutes. First, there were five minutes of anatomical scanning, followed by 2 × 21 minutes of testing with a short break in between the functional scans. Words and nonwords were presented on a 1024 × 768 pixel screen in white font on black background projected on a mirror inside the scanner. The font was “Arial” (50 pt). Words and nonwords were presented in random order to each subjects for 700 ms. After each stimulus, a blank screen with a fixation cross was presented. Presentation times of the fixation cross were jittered: 500 fixation crosses were presented for 2500 ms, 60 for 3200 ms, 25 for 7500 ms and 15 for 10500 ms. The experimental software used was Presentation from Neurobehavioral Systems (San Francisco, USA; http://www.neurobs.com/). Male or female names (30 in total; four to seven letters long) were randomly presented as catch trials during the experiment. Subjects were instructed to respond to the catch trials by pressing a button of an fMRI compatible button box (Cambridge Research Systems Ltd, Rochester, UK) whenever a name was presented during testing.

### Stimuli

The stimuli were the same as in^6^. Their GLA values were generated with the MROM as described in^5,85^ using a lexicon of 1025 monosyllabic three to five letter German words.

All 551 four-letter words were chosen from the CELEX database^86^ and a pool of 2000 nonwords was generated from these words by changing one, two, three, or four letters, excluding combinations that formed words. As a stable measure of global lexical activity (GLA), the average summed lexical activity generated across the first seven cycles of processing in the MROM was computed and transformed into z-values. 300 words and 300 nonwords were then selected so that the two resulting distributions were normal with significantly different means and equal variances. All 600 stimuli had four letters. Table 5 shows the stimulus characteristics for the different GLA levels for words and nonwords.

In addition, a correlation analysis of GLA with several linguistic variables for words and nonwords of the current study revealed several significant correlations showing that GLA as a single computational measure of the MROM reflects the linguistic properties of words and nonwords (neighborhood density: words = 0.78, nonwords = 0.75; summed frequency of neighbors: words = 0.36, nonwords = 0.31; number of higher frequency neighbors: words = 0.50, nonwords = 0.71; summed frequency of higher frequency neighbors: words = 0.34, nonwords = 0.31; bigram count: words = 0.67, nonwords = 0.74; summed bigram frequency: words = 0.40, nonwords = 0.31; and number of bigram neighbours: words = 0.33, nonwords = 0.49).

### Image acquisition

Functional and structural imaging was performed with a Siemens Tim Trio 3 Tesla scanner using a 32-channel head coil (Siemens, Erlangen, Germany). A gradient echo field map (TR 488 ms, TE 1 = 4.49 ms, TE 2 = 6.95 ms) and a high resolution (1 × 1 × 1.2 mm) structural scan with a T1 weighted MPRAGE sequence was acquired from each participant. The structural images were followed by two runs with 510 volumes each of functional images sensitive to BOLD contrast acquired with a T2* weighted gradient echo EPI sequence (TR = 2520 ms, TE = 33 ms, flip angle = 77°, number of slices = 36, slice thickness = 3 mm, 64 × 64 matrix, FOV = 192 mm). Six dummy scans were acquired at the beginning of each functional run before stimulus presentation. Low frequency noise was removed with a high-pass filter (128 s).

For preprocessing and statistical analysis, SPM8 software (http://www.fil.ion.ucl.ac.uk/spm/), running in a MATLAB 7.6 environment (Mathworks Inc., Natick MA, USA), was used. Functional images were realigned, unwarped, corrected for geometric distortions using the fieldmap of each participant and slice time corrected. The high resolution structural T1weighted image of each participant was processed and normalized with the VBM8 toolbox (http://dbm.neuro.uni-jena.de/vbm8) using default settings. Each structural image was segmented into gray matter, white matter and CSF and denoised and warped into MNI space by registering it to the DARTEL template provided by the VBM8 toolbox via the high-dimensional DARTEL^87^ registration algorithm. Based on these steps, a skull stripped version of each image in native space was created. To normalize functional images into MNI space, the functional images were co-registered to the skull stripped structural image and the parameters from the DARTEL registration were used to warp the functional images, which were resampled to 3 × 3 × 3 mm voxels and smoothed with a 8-mm FWHM Gaussian kernel.

### Imaging analysis

Statistical analysis was performed with a GLM two staged mixed effects approach. In the subject-specific first level model, each condition was modelled by convolving stick functions at its onsets with SPM8’s canonical hemodynamic response function and no time derivatives. On this subject-specific first level model conditions of interest were contrasted against the fixation baseline. These subject-specific contrast images were used for the second level group analysis. For statistical comparisons of words and nonwords against the fixation baseline a familywise error (FWE) corrected cluster threshold (p < 0.05) with a cluster extent of 25 was used. Direct comparisons of words and nonwords were thresholded at the cluster level at p < 0.05 corrected with false discovery rate (FDR), voxel level uncorrected p < 0.001. Furthermore, direct comparisons of levels of GLA for words and nonwords were thresholded at an uncorrected voxel level threshold of p < 0.005 and a cluster extent of 25 in order to allow potential activations in the medial temporal lobe to be included, given the lower signal to noise ratio in this region^88^. All stereotaxic coordinates for voxels with maximal z-values within activation clusters are reported in the MNI coordinate system.

## Supplementary information

Table S1. Brain regions showing a positive (GLApos) or negative (GLAneg) linear relationship of GLA and BOLD response (voxel-level uncorrected, p < .005, cluster extent threshold 25).
