# Supplementary material for: A model-guided dissociation between subcortical and cortical contributions to word recognition
Source: Sci Rep. 2019 Mar 14;9:4506. doi: 10.1038/s41598-019-41011-9 (PMC6418272; doi:10.1038/s41598-019-41011-9)
Supplement: Supplementary file 1 — Table S1. Brain regions showing a positive (GLApos) or negative (GLAneg) linear relationship of GLA and BOLD response (voxel-level uncorrected, p < .005, cluster extent threshold 25). [file 41598_2019_41011_MOESM1_ESM.docx]

A model-guided dissociation between subcortical and cortical contributions to word recognition.

Mario Braun*^1,2^, Martin Kronbichler^1,5,6^, Fabio Richlan^1^, Stefan Hawelka^1^, Florian Hutzler^1^, and Arthur M. Jacobs^2,3,4^

Centre for Cognitive Neuroscience, Universität Salzburg^1^, Allgemeine und Neurokognitive Psychologie, Freie Universität Berlin^2^, Center for Cognitive Neuroscience Berlin^3^, Dahlem Institute for Neuroimaging of Emotion^4^, Christian Doppler Klinik, Salzburg^5^, Paracelsus Medical University, Salzburg^6^

Supplementary Table S1

Table S1. Brain regions showing a positive (GLApos) or negative (GLAneg) linear relationship of GLA and BOLD response (voxel-level uncorrected, p < .005, cluster extent threshold 25).

| *Brain Region* | *BA* | *Hem* | *x* | *y* | *z* | *cluster size* | *Zmax* |
| --- | --- | --- | --- | --- | --- | --- | --- |
| **GLApos** | | | |  |  |  |  |
| Globus pallidus/Hippocampus | - | L | -15 | -7 | 1 | 40 | 3.99 |
|  | - | L | -12 | -16 | -11 |  |  |
|  | - | L | -18 | -22 | -11 |  |  |
| Hippocampus | - | R | 15 | -13 | -17 | 47 | 3.80 |
|  | - |  | 15 | -22 | -5 |  |  |
| Posterior cingulate cortex | - | L | -15 | -34 | 34 | 27 | 3.33 |
|  | - |  | -21 | -40 | 34 |  |  |
| Middle cingulate cortex | - | R | 15 | -16 | 37 | 27 | 3.18 |
|  | - |  | 18 | -13 | 25 |  |  |
|  | 23 |  | 9 | -16 | 31 |  |  |
|  | | | |  |  |  |  |
| **GLAneg** | | | |  |  |  |  |
| Precentral gyrus | 6 | L | -51 | -1 | 37 | 32 | 4.37 |
| Precentral gyrus | - | L | -24 | -10 | 43 | 121 | 3.89 |
| Superior frontal gyrus | 6 |  | -18 | -13 | 67 |  |  |
| Superior parietal lobule,  supramarginal gyrus, angular gyrus  postcentral gyrus | 2 | L | -42 | -40 | 55 | 160 | 3.86 |
|  | 7 |  | -30 | -58 | 58 |  |  |
|  | 3 |  | -36 | -37 | 64 |  |  |
| Precentral gyrus, pars opercularis | 48 | L | -54 | 2 | 19 | 25 | 3.81 |
| Supplementary motor cortex | 6 | L | -6 | -4 | 55 | 26 | 3.58 |
| Superior parietal lobule, angular gyrus | 7 | R | 27 | -67 | 49 | 30 | 3.27 |
|  | 7 |  | 24 | -61 | 37 |  |  |
